# Chronic stress promotes basal ganglia disinhibition by increasing the excitatory drive of direct-pathway neurons

**DOI:** 10.1016/j.ynstr.2023.100571

**Published:** 2023-09-22

**Authors:** Diana Rodrigues, Patricia Monteiro

**Affiliations:** a Life and Health Sciences Research Institute (ICVS), School of Medicine, University of Minho, Braga, Portugal; b ICVS/3B's-PT Government Associate Laboratory, Braga, Guimaraes, Portugal; c Department of Biomedicine - Experimental Biology Unit, Faculty of Medicine of the University of Porto (FMUP), Porto, Portugal

**Keywords:** Chronic stress, Dorsomedial striatum, Medial prefrontal cortex, Direct-pathway neurons, Hyperactivation

## Abstract

Chronic stress (CS) is a well-recognized triggering factor in obsessive-compulsive disorder (OCD) and Tourette's syndrome (TS), two neuropsychiatric disorders characterized by the presence of stereotypic motor symptoms. Planning and execution of motor actions are controlled by the dorsal striatum, a brain region that promotes or suppresses motor movement by activating striatal neurons from the direct- or indirect-pathway, respectively. Despite the dorsal striatum being affected in motor disorders and by CS exposure, how CS affects the two opposing pathways is not fully understood. Here, we report that CS in mice selectively potentiates the direct-pathway, while sparing the indirect-pathway. Specifically, we show that CS both increases excitation and reduces inhibition over direct-pathway neurons in the dorsomedial striatum (DMS). Furthermore, inhibitory interneurons located in the DMS also display reduced excitatory drive after chronic stress, thus amplifying striatal disinhibition. Altogether, we propose a model where both increased excitatory drive and decreased inhibitory drive in the striatum causes disinhibition of basal ganglia's motor direct pathway - a mechanism that might explain the emergence of motor stereotypies and tic disorders under stress.

## 1 Introduction

The basal ganglia are a group of interconnected subcortical nuclei that include the striatum, pallidum, subthalamic nucleus, and substantia nigra (Groenewegen, 2003). The striatum is the entryway to the basal ganglia and is the source of the direct- and indirect-pathways, two basal ganglia circuits that are critical for the control of intended motor actions (Graybiel, 2008; Hauber and Schmidt, 1994; Kreitzer, 2009). The direct-pathway circuit originates from medium spiny neurons (MSNs) in the striatum that express dopamine receptor type 1 (D1-MSNs), whereas the indirect-pathway originates from striatal MSNs that express dopamine receptor type 2 (D2-MSNs). These basal ganglia pathways control movement in opposing ways: activation of the direct pathway promotes motor actions while activation of the indirect pathway inhibits motor actions (Gerfen and Surmeier, 2011; Kreitzer and Malenka, 2008).

At the cellular level, MSNs’ output is tightly regulated by local GABAergic interneurons that provide strong inhibitory control, such as parvalbumin-positive (PV) interneurons. Both cellular populations, MSNs and PV, receive glutamatergic/excitatory inputs from upstream cortical neurons (Choi et al., 2019; Graybiel et al., 1994; Klug et al., 2018; Kress et al., 2013; Landry et al., 1984; Lovinger and Tyler, 1996; Monteiro et al., 2018; Reiner et al., 2003; Shepherd, 2004; Tepper et al., 2004, 2008; Wilson, 1987). These cortical neurons can recruit either D1 direct-pathway MSNs or D2 indirect-pathway MSNs, respectively promoting or suppressing the execution of motor actions (Kreitzer and Malenka, 2008).

Early clinical work suggests that striatal dysfunction might be central to the emergence of obsessive-compulsive disorder (OCD) (Graybiel and Rauch, 2000; Maia et al., 2008) and Tourette's syndrome (TS) (Hienert et al., 2018), two neuropsychiatric disorders characterized by stereotypic unwanted motor actions. Interestingly, striatal dysfunction is also observed after exposure to chronic stress (CS), and CS itself is known to trigger and exacerbate motor symptoms in OCD and TS (Godar and Bortolato, 2017; Sousa-Lima et al., 2019). Despite this strong link between striatum, stress, OCD, and TS, an explanation at the cell-circuit level for how stress might mechanistically be able to trigger motor symptoms, is still elusive.

Previous work from our group has shown that CS leads to striatal disinhibition causing increased MSN firing activity and increased motor locomotion in stressed mice (Rodrigues et al., 2022). Here, we demonstrate that CS in mice selectively facilitates the striatal direct-pathway, a pathway that promotes motor output, thus providing a mechanistic explanation for the emergence of motor stereotypies and tic disorders under chronic stress. By increasing excitatory drive over striatal D1 MSNs while simultaneously reducing excitatory drive over striatal PV inhibitory interneurons, CS promotes the activation of basal ganglia's direct-pathway, a mechanism highly relevant for explaining stress-triggered motor symptoms.

## 2 Materials and methods

### 2.1 Animals

All animal procedures were approved by local authorities Direção Geral de Alimentação e Veterinária (ID: DGAV 8519) and the Ethics Subcommittee for the Life Sciences and Health (SECVS) of the University of Minho (ID: SECVS 01/18) and performed in accordance with European Community Council Directives (2010/63/EU) and the Portuguese law DL Nº 113/2013 for the care and use of laboratory animals. Animals were housed in a temperature-controlled room (22 °C; 55% humidity) under a 12-h light/dark cycle (lights ON at 8 a.m.) with *ad libitum* access to water and food (4RF21, Mucedola).

*Drd1a-tdTomato* (Shuen et al., 2008), *Drd2-EGFP* (Gong et al., 2003)*,* and *Pvalb-tdTomato* (Kaiser et al., 2016) mice were bred on a pure C57BL/6 background and maintained as separate transgenic lines. Heterozygous male mice were randomly assigned to the CS group with corresponding littermates assigned to the control (non-stressed) group and housed separately by the experimental group. For the social defeat paradigm, 3–12 months old male CD1 mice from Charles River Laboratories were used as residents. CD1 mice were individually housed to increase their territorial status, and bedding was not changed during the stress protocol.

### 2.2 Chronic unpredictable stress

Chronic unpredictable stress protocol was performed as described previously (Rodrigues et al., 2022). Briefly, 5 weeks old male mice were exposed once a day to one of three random stressors: forced swimming, restraint, or social defeat. During forced swimming, mice were placed inside a 20 cm diameter cylinder half-filled with 24 ± 1 °C water and forced to swim for 5 min. During restraint protocol, mice were restrained for 15 min inside a 50 mL falcon tube containing breathing holes. The social defeat protocol was based on the resident-intruder paradigm (Golden et al., 2011). The intruder mouse was placed inside the resident mouse's cage and allowed to interact with the resident for a maximum of 5 min or until being attacked and defeated by the resident (as indicated by fleeing, freezing, or submissive behaviour). Afterwards, the intruder was separated from the resident but kept inside the resident's cage for 30 min inside an acrylic enclosure that allowed visual, auditory, and olfactory contact but prevented further direct physical attack. Stressors were randomly distributed throughout 21 days and arbitrarily scheduled in terms of daytime, to prevent the animals from predicting and adapting to the stressor. In all cohorts, mice were exposed to the same order and schedule of stressors. This paradigm was conceived to maximize unpredictability and to better mimic the variability of stressors encountered in daily life (Amat et al., 2005; Atrooz et al., 2021; Dias-Ferreira et al., 2009).

### 2.3 Retro-orbital injection

For morphological studies, 3 weeks old *Drd1a-tdTomato* mice were injected with AVV9.hSyn.eGFP.WPRE.bGH virus (Penn Vector Core, University of Pennsylvania) into the retro orbital sinus as described previously (Yardeni et al., 2011). Briefly, mice were anaesthetized with isoflurane and 1 μL of the virus with a titre of 1.32 x 10^14^ genome copies (GC) ml^−1^ was injected into the retro-orbital sinus cavity. Two weeks after retro-orbital injection, mice were randomly assigned into control (Ctrl, *n* = 4) or stressed (CS, *n* = 4) groups, followed by 21 days of chronic unpredictable stress protocol in the latter case.

### 2.4 Immunohistochemistry and morphological studies

Mice were transcardially perfused with saline followed by 4% PFA. Brains were dissected out, post-fixed by overnight immersion in 4% PFA, and then transferred to a 30% sucrose in PBS solution for 24 h immersion at 4 °C. After that, the brains were embedded in OCT (Bio-Optica) and serially cut in a cryostat (Leica Microsystems). 30-μm-thick sagittal sections were used for strain validation and 100-μm-thick coronal sections were used for morphological studies.

For transgenic strain validation, *Drd1a-tdTomato* and *Drd2-EGFP* native fluorescence was used for imaging. For morphological studies, striatal brain sections were washed three times for 10 min with PBS and placed in citrate buffer at 80 °C for 20 min. After that, the brain sections were allowed to cool down at RT for 20 min and then washed 3 times for 10 min with PBS. Brain sections were permeabilized twice with 0.3% Triton X-100 (Sigma–Aldrich) in PBS for 10 min at RT. After washing 3 times in PBS for 10 min, brain sections were blocked using 15%NGS, 5%BSA, 0.2% Triton-x for 1hr at RT. Blocked sections were then incubated overnight with primary antibody for GFP (Mouse #MAB3580, Millipore, 1:1000) diluted in blocking buffer. Following primary antibody incubation, brain sections were washed three times for 10 min in PBS and incubated with secondary antibody (488-Goat anti-mouse IgG, Invitrogen, 1:1000), for 2 h at RT. Next, brain sections were washed three times for 10 min with PBS, stained for DAPI (D9542-1 MG Sigma–Aldrich) for 3 min at RT, and mounted on Superfrost slides (Thermo Scientific) using Shandon™ Immu-Mount™ mounting medium (Thermo Scientific).

Image acquisition was performed using Olympus confocal microscope (FV1000, Olympus) and blinded to the experimental groups (control versus chronic stress). Serial optical sections (z-stacks) were acquired with a 40× oil immersion objective for morphological studies. Isolated neurons with non-overlapping dendritic trees were chosen, and z-series of the same neuron were stitched together using FV10-ASW 4.2 Viewer software (Olympus). Neuronal arbor reconstruction and analysis were carried out using the Simple Neurite Tracer plugin in ImageJ software. No correction was applied for tissue shrinkage during fixation.

### 2.5 Electrophysiology slice recordings

Whole-cell patch clamp recordings were used to measure synaptic currents and intrinsic properties in striatal and cortical neurons. Acute slices from control and chronic stressed mice were used for all experiments. Animals were deeply anaesthetized with avertin (tribromoethanol; 20 mg/mL; Sigma–Aldrich) with a dose of 0.5 mg/g body weight by intraperitoneal injection and subsequently checked for lack of paw withdrawal reflexes before being transcardially perfused with 15–20 mL of carbogenated N-methyl-ꓓ-glucamine (NMDG)-based artificial cerebrospinal fluid (aCSF) solution (mM): 92 NMDG, 2.5 KCl, 1.2 NaH_2_PO_4_, 30 NaHCO_3_, 20 HEPES, 25 glucose, 5 sodium ascorbate, 2 thiourea, 3 sodium pyruvate, 10 MgSO_4_.7H_2_O, and 0.5 CaCl_2_.2H_2_O, (7.2–7.4 pH and 300–310 mOsm/L). After decapitation, brains were rapidly removed and placed in the same carbogenated NMDG solution for slice preparation. A Vibratome VT1000S (Leica Microsystems) was used to prepare 300-μm-thick striatum coronal slices. Slices were then incubated at 32–34 °C for 11 min in carbogenated NMDG solution and transferred to a holding chamber (Brain Slice Keeper 4-Quad, Automate Scientific Inc.) filled with carbogenated aCSF solution (mM): 119 NaCl, 2.5 KCl, 1.2 NaH_2_PO_4_, 24 NaHCO_3_, 12.5 glucose, 2 MgSO_4_.7H_2_O and 2 CaCl_2_.2H_2_O (7.2–7.4 pH and 300–310 mOsm/L). Slices were allowed to recover at least 1 h at RT before recordings. Recordings were made at RT (22–25 °C) and carbogenated aCSF was perfused at approximately 3 mL/min. Patch pipettes were pulled from borosilicate glass with filament (GB150F–8P, Science Products) on a P1000 horizontal puller (Sutter Instruments) with a typical resistance of 2–5 MΩ when backfilled with the internal solution. For current-clamp recordings of intrinsic properties, patch pipettes were filled with KGlu internal solution containing (in mM): 131 potassium gluconate, 17.5 KCl, 9 NaCl, 1 MgCl_2_.6H_2_O, 10 HEPES, 1.1 EGTA, 2 MgATP and 0.2 NaGTP (pH adjusted to 7.3 with KOH and osmolarity adjusted to 300 mOsm/L with sucrose). For voltage-clamp recordings of miniature inhibitory postsynaptic currents (mIPSCs), patch pipettes were filled with CsCl internal solution containing (in mM): 103 CsCl, 12 CsOH, 12 methanesulfonic acid, 5 TEA-Cl, 10 HEPES, 4 MgATP, 0.3 NaGTP, 10 phosphocreatine, 0.5 EGTA, 5 lidocaine N-ethylchloride, and 4 NaCL (pH adjusted to 7.3 with KOH and osmolarity adjusted to 300 mOsm/L with K_2_SO_4_). During mIPSC recordings, slices were perfused with carbogenated aCSF in the presence of 50 μM DL-AP5 (dl-2-amino-5-phosphonovaleric acid, Tocris), 10 μM NBQX (2,3-Dioxo-6-nitro-1,2,3,4-tetrahydrobenzo[f]quinoxaline-7-sulfonamide, Tocris) and 1 μM tetrodotoxin (Tocris). For miniature excitatory postsynaptic currents (mEPSC) recordings, patch pipettes were filled with a CsGlu internal solution containing (in mM): 110 CsOH, 110 ꓓ-gluconic acid, 15 KCl, 4 NaCl, 5 TEA-Cl, 20 HEPES, 0.2 EGTA, 5 lidocaine N-ethylchloride, 4 MgATP, and 0.3 NaGTP (pH adjusted to 7.3 with KOH and osmolarity adjusted to 300 mOsm/L with K_2_SO_4_). During mEPSC recordings, slices were perfused with carbogenated aCSF in the presence of 100 μM picrotoxin (Tocris) and 1 μM tetrodotoxin (Tocris). Both mIPSC and mEPSC recordings were performed at −70 mV holding potential. Intrinsic properties were obtained from a series of hyperpolarizing and depolarizing current and voltage step injections. Input resistance was calculated with a −100 pA hyperpolarizing step from the resting membrane potential, as well as from a linear fit to a voltage-current plot. To measure the overall charge transfer across the membrane, the synaptic drive was calculated for each recorded neuron by multiplying the mPSC average frequency by the mPSC average amplitude. Whole-cell patch-clamp recordings were obtained after seal rupture and internal equilibrium, under a BX-51WI microscope (Olympus) equipped with fluorescence and infrared differential interference contrast (IR-DIC). Data were acquired using a Digidata 1440A and a MultiClamp 700B amplifier (Molecular Devices, USA). The signals for voltage-clamp recordings were low-pass filtered at 2 kHz and digitized at 10 kHz. For current-clamp recordings, the bridge balance was adjusted, and the theoretical liquid junction potential was not corrected. In all recordings, D1-MSNs, D2-MSNs, and PV interneurons were identified based on native fluorescence and pyramidal cells were identified based on their morphology. Only cells with series-resistance values < 25 MΩ were recorded. Intrinsic properties, mIPSC, and mEPSC were analysed using pClamp (Clampfit; Axon Instruments) and Minianalysis software (Synaptosoft).

### 2.6 Statistical analysis

All statistical analyses were performed using Prism (GraphPad Software Inc.). Data are expressed as mean ± SEM. Significance was determined at the level of p < 0.05. Non-normal distributions were considered for all the data sets, regardless of variance and sample size. Pairwise comparisons were performed using a Mann-Whitney test for unpaired data and Wilcoxon signed-rank test for paired data comparisons. Further details on particular analyses are shown in Sup.Table 1.

## 3 Results

### 3.1 Chronic stress causes morphological changes in striatal neurons from the direct pathway only

**Fig. 1:**
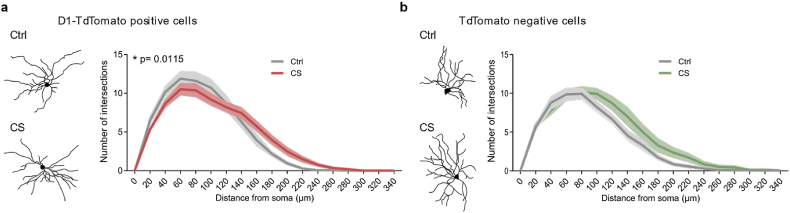
**Chronic stress impacts the dendritic morphology of striatal D1 but not D2 neurons**. (a) Morphometric analysis of Drd1a-tdTomato positive cells in the dorsomedial striatum (DMS) region of Drd1a-tdTomato transgenic mice from control (Ctrl; grey) and chronic stress (CS; red) mice. Representative images of neurons from control and stressed animals are shown on the left. Right panels show the number of dendritic branches (arbor complexity) correlated with distance from the cell body (soma). Drd1a-tdTomato positive cells Ctrl *n* = 21, CS *n* = 18. (b) Morphometric analysis of Drd1a-tdTomato negative cells (right) in the DMS of Drd1a-tdTomato transgenic mice from control (Ctrl; grey) and chronic stress (CS; green) mice. Representative images of neurons from control and stressed animals are shown on the left. Right panels show the number of dendritic branches (arbor complexity) correlated with distance from the cell body (soma). Drd1a-tdTomato negative cells *n* = 14 Ctrl and *n* = 14 CS. Shaded error bars represent SEM. All data from 4 control and 4 stressed mice; Two-way ANOVA with multiple comparisons. Statistical details are shown in Sup.Table1. (For interpretation of the references to colour in this figure legend, the reader is referred to the Web version of this article.)

Striatal neurons comprise two major opposing cellular populations of medium spiny neurons (MSNs): D1 direct- and D2 indirect-pathway MSNs, that respectively promote and suppress motor actions (Gerfen and Surmeier, 2011; Kreitzer and Malenka, 2008). It has been shown that chronic stress increases the overall firing activity of MSNs (Friedman et al., 2017; Rodrigues et al., 2022). However, it is still unknown whether stress impacts differentially the two opposing MSN pathways. Here, we start by asking whether CS could be differently affecting D1 and D2 neurons and whether an effect could be observed at the morphological level. However, because D1 and D2 neurons are indistinguishable in terms of gross morphology, we had to use *Drd1a-tdTomato* transgenic mice and then apply a viral-based strategy previously developed by us to sparsely label cells with eGFP through retro-orbital injections of AVV.eGFP (Zhang et al., 2016). Such fluorescent labelling strategy allows colocalization between the eGFP signal (from the virus) and the *tdTomato* signal present in D1 neurons from *Drd1a-tdTomato* mice. Using this sparse labelling viral approach, we found that stressed mice exhibited morphological changes in D1-MSNs (Fig. 1a; *p* = 0.0115; Sup.Table1) but no significant changes in neighbouring putative D2-MSNs (eGFP-positive but *tdTomato*-negative MSNs), from the same region in the same mice (Fig. 1b; *p* = 0.2052; Sup.Table1). These morphological changes in D1-MSNs, suggest that exposure to CS preferentially impacts striatal neurons from the direct pathway.

### 3.2 Excitatory synaptic transmission is increased onto direct pathway neurons only

**Fig. 2:**
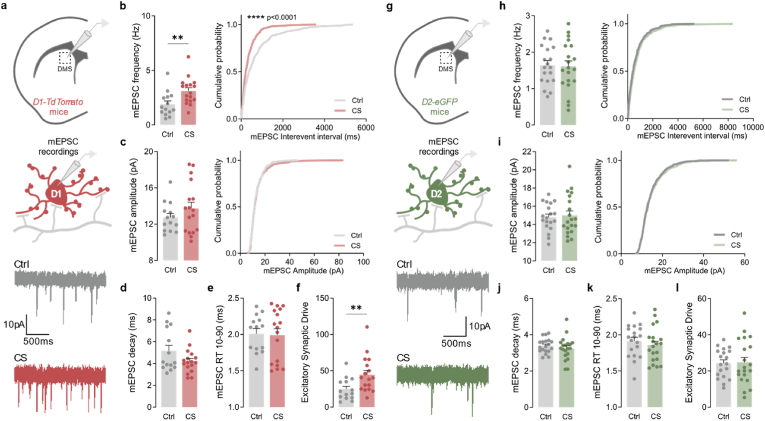
**Chronic stress increases excitatory synaptic transmission onto D1 but not D2 neurons**. (a) Example traces of miniature excitatory postsynaptic currents (mEPSC) recorded from fluorescently labeled D1-MSNs in dorsomedial striatum region (DMS) of control (Ctrl; grey) and stressed (CS; red) mice. (b) Summary bar graphs (Ctrl *n* = 14 and CS *n* = 17 cells; **p = 0072) and cumulative probability curves (30 events per cell; ****p < 0.0001) show increased mEPSC frequency in D1-MSNs from stressed mice. (c) Summary bar graphs (Ctrl *n* = 14 and CS *n* = 17 cells) and cumulative probability curves (30 events per cell) show similar mEPSC amplitude in D1-MSNs from stressed mice. (d,e) Summary bar graphs (Ctrl *n* = 14 and CS *n* = 17 cells) show no significant differences in the kinetics of mEPSC recorded from D1-MSNs in stressed mice. (f) Summary bar graphs (Ctrl *n* = 14 and CS *n* = 17 cells; **p = 0.01) show increased excitatory synaptic drive, defined as mEPSC frequency x mEPSC amplitude per individual neuron, in D1-MSNs from stressed mice. (g) Example traces of miniature excitatory postsynaptic currents (mEPSC) recorded from fluorescently labeled D2-MSNs in the DMS of control (Ctrl; grey) and stressed (CS; green) mice. (h) Summary bar graphs (Ctrl *n* = 19 and CS *n* = 20 cells) and cumulative probability curves (20 events per cell) show similar mEPSC frequency in D2-MSNs from stressed mice. (i) Summary bar graphs (Ctrl *n* = 19 and CS *n* = 20 cells) and cumulative probability curves (20 events per cell) show similar mEPSC amplitude in D2-MSNs from stressed mice. (j,k) Summary bar graphs (Ctrl *n* = 19 and CS *n* = 20 cells) show no significant differences in the kinetics of mEPSC recorded from D2-MSNs in stressed mice. (l) Summary bar graphs (Ctrl *n* = 19 and CS *n* = 20 cells) show no alterations on the excitatory synaptic drive, defined as mEPSC frequency x mEPSC amplitude per individual neuron, in D2-MSNs of stressed mice. All bar graphs are mean ± SEM; Two-sided Welch's unpaired *t*-test (b-f, h-l), and Kolmogorov-Smirnov test (b-c curves, h-I curves). Statistical details are shown in Sup.Table1. (For interpretation of the references to colour in this figure legend, the reader is referred to the Web version of this article.)

To further investigate whether the morphological changes observed in striatal neurons from the direct pathway were mirrored by functional changes, we performed whole-cell patch-clamp recordings of synaptic currents. Again, because the striatum contains two major opposing cellular populations of neurons (D1 direct- and D2 indirect-pathway MSNs (Kreitzer and Malenka, 2008)), we used transgenic mouse lines to achieve cell-type specific fluorescent labelling of both MSN populations (Fig. S1). In line with our previous morphological observation of stress impact over D1-MSNs, miniature excitatory postsynaptic currents (mEPSC) recorded from striatal D1-MSNs (Fig. 2a–f) revealed increased frequency (Ctrl 1.902 ± 0.290, CS 3.110 ± 0.284; *p* = 0.0076) (Fig. 2b) and increased excitatory synaptic drive (Ctrl 24.658 ± 3.934, CS 44.676 ± 5.785; *p* = 0.0100) (Fig. 2f) in stressed mice. In contrast, mEPSC recorded from striatal D2-MSNs did not significantly differ between stressed mice and littermate controls (Fig. 2g-l and Sup.Table1). Taken together, these findings indicate that CS has circuit-selective effects in the striatum, specifically increasing excitatory synaptic transmission over D1 direct-pathways neurons while sparing D2 indirect-pathway neurons.

### 3.3 Inhibitory synaptic transmission is reduced onto direct pathway neurons only

**Fig. 3:**
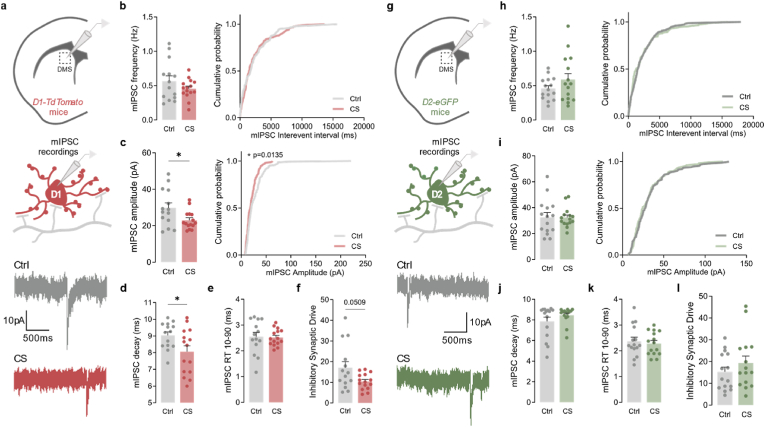
**Chronic stress decreases inhibitory synaptic transmission in D1 neurons**. (a) Example traces of miniature inhibitory postsynaptic currents (mIPSC) recorded from tdTomato labeled D1-MSNs in dorsomedial striatum region (DMS) of control (Ctrl; grey) and stressed (CS; red) mice. (b) Summary bar graphs (Ctrl *n* = 14 and CS *n* = 15 cells) and cumulative probability curves (10 events per cell) show similar mIPSC frequency in D1-MSNs from stressed mice. (c) Summary bar graphs (Ctrl *n* = 14 and CS *n* = 15 cells; *p = 0.0369) and cumulative probability curves (10 events per cell; *p = 0.0456) show reduced mIPSC amplitude in D1-MSNs from stressed mice. (d,e) Summary bar graphs (Ctrl = 14 and CS n = 15 cells) show a decrease in mIPSC decay kinetics (*p = 0.0262) and no significant differences in rise time (RT) in D1-MSNs from stressed mice. (f) Summary bar graphs (Ctrl *n* = 14 and CS *n* = 15 cells) show a clear tendency towards decreased inhibitory synaptic drive, defined as mIPSC frequency x mIPSC amplitude per individual neuron, in D1-MSNs from stressed mice. (g) Example traces of miniature inhibitory postsynaptic currents (mIPSC) recorded from GFP labeled D2-MSNs in the DMS of control (Ctrl; grey) and stressed (CS; green) mice. (h) Summary bar graphs (Ctrl *n* = 15 and CS *n* = 15 cells) and cumulative probability curves (10 events per cell) show similar mIPSC frequency in D2-MSNs from stressed mice. (i) Summary bar graphs (Ctrl *n* = 15 and CS *n* = 15 cells) and cumulative probability curves (10 events per cell) show similar mIPSC amplitude in D2-MSNs from stressed mice. (j,k) Summary bar graphs (Ctrl *n* = 15 and CS *n* = 15 cells) show no significant differences in the kinetics of mIPSC recorded from D2-MSNs in stressed mice. (l) Summary bar graphs (Ctrl *n* = 15 and CS *n* = 15 cells) show no differences in inhibitory synaptic drive, defined as mIPSC frequency x mIPSC amplitude per individual neuron, in D2-MSNs from stressed mice. All bar graphs are mean ± SEM; Two-sided Welch's unpaired *t*-test (b-f, h-l), and Kolmogorov-Smirnov test (b-c curves, h-I curves). Statistical details are shown in Sup.Table 1. (For interpretation of the references to colour in this figure legend, the reader is referred to the Web version of this article.)

Given the increased excitatory transmission detected after CS, we next asked whether a proportional increase could also be observed in inhibitory transmission as a compensatory mechanism to normalize the final E/I ratio (excitatory/inhibitory ratio), thus maintaining homeostasis. To answer this question, we recorded whole-cell miniature inhibitory postsynaptic currents (mIPSC) from D1-and D2-MSNs (Fig. 3a, g). Compared to control littermates, stressed mice exhibited decreased mIPSC amplitude in D1-MSNs (Ctrl 29.833 ± 2.601, CS 23.089 ± 1.315; *p* = 0.0369) (Fig. 3c), without frequency changes (Ctrl 0.565 ± 0.075, CS 0.455 ± 0.037; *p* = 0.2180) (Fig. 3b). Furthermore, stressed mice also presented a clear trend for a reduced inhibitory synaptic drive in D1-MSNs (Ctrl 17.047 ± 2.855, CS 10.444 ± 1.000; *p* = 0.0509) (Fig. 3f). In terms of mIPSC kinetics, D1-MSNs displayed faster mIPSC decay in stressed mice (Ctrl 9.036 ± 0.205, CS 8.063 ± 0.349; *p* = 0.0262) (Fig. 3d), without differences in rise time (Ctrl 2.545 ± 0.167, CS 2.519 ± 0.084; *p* = 0.8945) (Fig. 3e). Once again, no significant changes were detected in D2-MSNs from stressed mice (Fig. 3g-l and Sup.Table1). These findings, together with our previous data, suggest that CS has profound differential effects over striatum pathways, potentiating the direct pathway by increasing excitation as well as reducing inhibition over striatal D1-MSNs.

### 3.4 Chronic stress reduces excitatory drive onto striatal PV interneurons

**Fig. 4:**
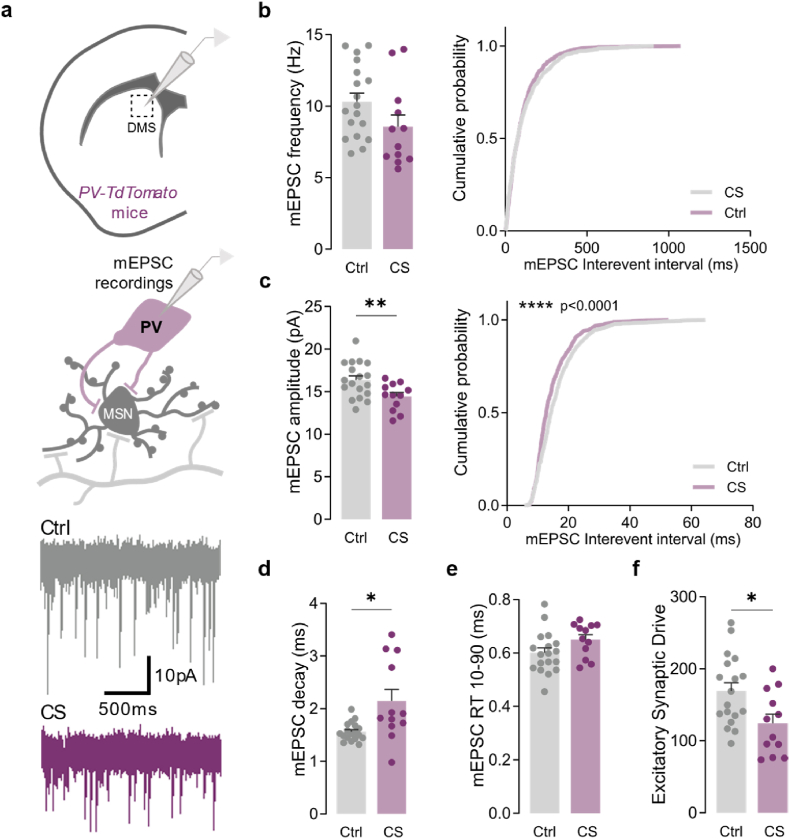
**Chronic stress decreases excitatory synaptic transmission strength onto striatal PV interneurons**. (a) Example traces of miniature excitatory postsynaptic currents (mEPSC) recorded from tdTomato labeled parvalbumin (PV) interneurons in dorsomedial striatum region (DMS) of control (Ctrl; grey) and stressed (CS; purple) mice. (b) Summary bar graphs (Ctrl *n* = 18 and CS *n* = 12 cells) and cumulative probability curves (50 events per cell) show a clear tendency towards decreased mEPSC frequency in PV interneurons from stressed mice. (c) Summary bar graphs (Ctrl *n* = 18 and CS *n* = 12 cells; **p = 0.0080) and cumulative probability curves (50 events per cell; ****p < 0.0001) show reduced mEPSC amplitude in PV interneurons from stressed mice. (d,e) Summary bar graphs (Ctrl *n* = 18 and CS *n* = 12 cells) show a significantly slower mEPSC decay kinetics (*p = 0.0234) and no differences in rise time (RT; p = 0.6009) in PV interneurons from stressed mice. (f) Summary bar graphs (Ctrl *n* = 18 and CS *n* = 12 cells; *p = 0.0143) show a decreased excitatory synaptic drive, defined as mEPSC frequency x mEPSC amplitude per individual interneuron, in PV cells from stressed mice. All bar graphs are mean ± SEM Two-sided Welch's unpaired *t*-test (b–f), and Kolmogorov-Smirnov test (b-c curves). Statistical details are shown in Sup.Table1. (For interpretation of the references to colour in this figure legend, the reader is referred to the Web version of this article.)

**Fig. 5:**
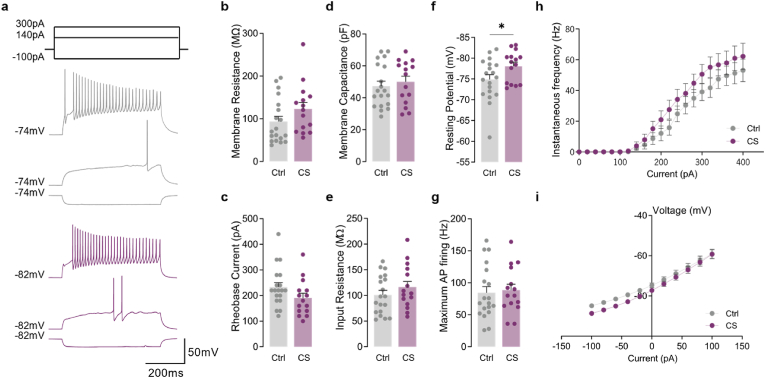
**PV interneurons have more hyperpolarized resting membrane potential after CS**. (a) Representative current-clamp recordings from tdTomato labeled PV interneurons in the DMS of control (Ctrl; grey) and chronic stress (CS; purple) mice. (b) Resting membrane potential (Ctrl *n* = 19 and CS *n* = 15 cells; *p = 0.0416) is significantly more hyperpolarized in PV interneurons from stressed mice. (c) Membrane capacitance (Ctrl *n* = 19 and CS *n* = 15 cells) is not significantly altered in PV interneurons from stressed mice. (d) Rheobase current (Ctrl *n* = 19 and CS *n* = 15 cells) is not significantly altered in PV interneurons from stressed mice. (e) Membrane resistance (Ctrl *n* = 19 and CS *n* = 15 cells) showed no significant alterations in PV interneurons from stressed mice. (f) Input resistance (Ctrl *n* = 19 and CS *n* = 15 cells) is not significantly different in PV interneurons from stressed mice. (g) Maximum action potential (AP) firing (Ctrl *n* = 19 and CS *n* = 15 cells) is not significantly altered in PV interneurons from stressed mice. (h) Action potential firing frequency (Hz) plotted as a function of injected current steps (Ctrl *n* = 19 and CS *n* = 15 cells). (i) Current-voltage plots (Ctrl *n* = 19 and CS *n* = 15 cells) recorded from PV interneurons showed no differences in stressed mice. All bar graphs are mean ± SEM; Two-sided Welch's unpaired *t*-test (b–g), and two-way repeated-measures ANOVA (h–i). Statistical details are shown in Sup.Table1. (For interpretation of the references to colour in this figure legend, the reader is referred to the Web version of this article.)

Disruption of local connectivity between PV interneurons and striatal MSNs has been previously suggested in OCD, TS, and dystonia (Burguière et al., 2015; Gernert et al., 2000; Kalanithi et al., 2005; Monteiro and Feng, 2016a; Xu et al., 2016). Interestingly, striatal PV interneurons are more likely to target the D1 direct pathway neurons rather than D2 indirect pathway neurons, making feedforward inhibition a more prominent feature of the direct pathway (Gittis et al., 2010). Therefore, this raises the possibility that excitatory synaptic drive over PV interneurons could also be affected by CS exposure. In fact, recent work has suggested that CS could be “disconnecting” striatal PV interneurons from excitatory cortical input (indirectly weakening their inhibitory control over MSNs) (Friedman et al., 2017). To test this hypothesis, we recorded AMPA-mediated excitatory transmission directly from PV interneurons using targeted whole-cell recordings in control and stressed *Pvalb-tdTomato* mice (Fig. 4a–f). Compared to controls, PV interneurons from stressed mice exhibited a remarkable decrease in mEPSC amplitude (Ctrl 16.381 ± 0.479, CS 14.439 ± 0.446; *p* = 0.0080) (Fig. 4c) and a trend for reduced frequency (Ctrl 10.316 ± 0.580, CS 8.571 ± 0.791; *p* = 0.1012) (Fig. 4b), confirming the hypothesis of weakened excitatory drive after CS exposure (Ctrl 169.435 ± 11.191, CS 124.439 ± 12.124; *p* = 0.0143) (Fig. 4f). mEPSC recorded from PV interneurons in stressed mice also displayed slower decay kinetics (Ctrl 1.562 ± 0.040, CS 2.142 ± 0.214; *p* = 0.0234) (Fig. 4d), with no changes in rise time (Ctrl 0.601 ± 0.018, CS 0.651 ± 0.018; *p* = 0.0660) (Fig. 4e). To further understand whether such synaptic changes were accompanied by changes in intrinsic excitability, we also recorded active and passive membrane properties from striatal PV interneurons after CS (Fig. 5). Results revealed that PV interneurons from stressed mice displayed more hyperpolarized resting membrane potential (Ctrl −74.891 ± 1.130, CS -78.091 ± 0.929; *p* = 0.0416) (Fig. 5f), a mechanism by which CS could be further decreasing PV inhibitory efficiency over D1-MSNs. Intrinsic properties recorded from D1-MSNs revealed no differences between control and stressed mice (Fig. S2). Altogether, our data support the hypothesis that CS selectively promotes the activation of the striatal direct pathway by further releasing D1-MSNs from the inhibitory control of local PV interneurons.

### 3.5 Chronic stress alters glutamatergic synaptic transmission in layer 5/6 of infralimbic and prelimbic cortices

**Fig. 6:**
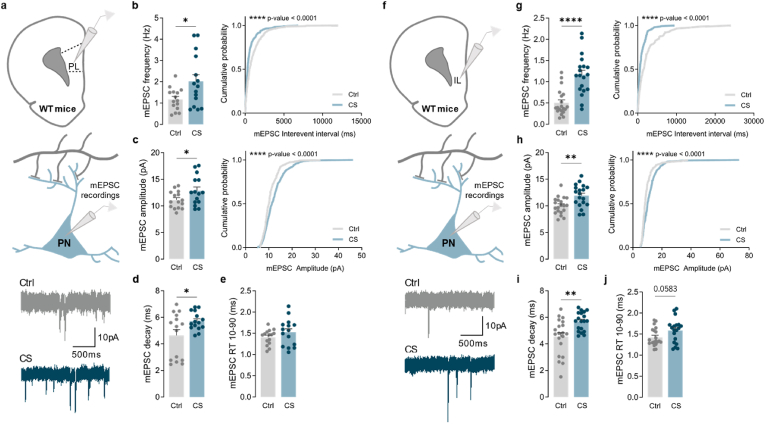
**Chronic stress increases excitatory synaptic transmission onto pyramidal cells from L5/6 of prelimbic and infralimbic cortices**. (a) Example traces of miniature excitatory postsynaptic currents (mEPSC) recorded from layer 5/6 (L5/6) pyramidal neurons (PN) in the prelimbic (PL) subregion, in control (Ctrl; grey) and stressed (CS; blue) mice. (b) Summary bar graphs (Ctrl *n* = 15 and CS *n* = 15 cells; *p = 0.0250) and cumulative probability curves (20 events per cell; ****p < 0.0001) show increased mEPSC frequency in L5/6 pyramidal neurons in the PL of stressed mice. (c) Summary bar graphs (Ctrl *n* = 15 and CS *n* = 15 cells; *p = 0.0470) and cumulative probability curves (20 events per cell; ****p < 0.0001) show enhanced mEPSC amplitude in L5/6 pyramidal neurons in the PL of stressed mice. (d,e) Summary bar graphs (Ctrl *n* = 15 and CS *n* = 15 cells; *p = 0.0348) show slower mEPSC decay kinetics and no significant differences in the rise time (RT) in L5/6 pyramidal neurons in the PL from stressed mice. (f) Example traces of mEPSC recorded from L5/6 pyramidal neurons in the infralimbic (IL) subregion, in control (Ctrl; grey) and stressed (CS; blue) mice. (g) Summary bar graphs (Ctrl *n* = 20 and CS *n* = 19 cells; ****p < 0.0001) and cumulative probability curves (8 events per cell; ****p < 0.0001) show remarkably increased mEPSC frequency in L5/6 pyramidal neurons in the IL from stressed mice. (h) Summary bar graphs (Ctrl *n* = 20 and CS *n* = 19 cells; **p = 0.0033) and cumulative probability curves (8 events per cell; ****p < 0.0001) show increased mEPSC amplitude in L5/6 pyramidal neurons in the IL from stressed mice. (i,j) Summary bar graphs (Ctrl *n* = 20 and CS *n* = 19 cells) show slower mEPSC decay kinetics (**p = 0.0024) and a clear tendency towards increased rise time (RT; *p* = 0.0583) in L5/6 pyramidal neurons in the IL from stressed mice. All bar graphs are mean ± SEM; Two-sided Welch's unpaired *t*-test (b-e, g-j), and Kolmogorov-Smirnov test (b-c curves, g-h curves). Statistical details are shown in Sup.Table1. (For interpretation of the references to colour in this figure legend, the reader is referred to the Web version of this article.)

The dorsomedial striatum (DMS) receives broad afferent excitatory inputs from the medial pre-frontal cortex (mPFC), a circuit that is critical for motor and action planning (Pennartz et al., 2009) and that seems to be impaired in stress-related disorders (Friedman et al., 2017; Nagarajan et al., 2018; Welch et al., 2007). To test whether the increased excitatory synaptic transmission observed in our recordings could arise from a dysfunctional cortical circuitry, we recorded mEPSC from the IL and PL subregions of mPFC that project to DMS. Since mPFC is a layer-organized structure, and prefrontal neurons project to the striatum in a layer-based distribution (Gabbott et al., 2005; Hunnicutt et al., 2016; Kim et al., 2017; Kupferschmidt et al., 2017; Murugan et al., 2017; Otis et al., 2017), we collected whole-cell recordings from layer 2/3 (L2/3) and layer 5/6 (L5/6) pyramidal neurons, the only output layers of mPFC. Results revealed that CS had a tremendous impact on excitatory synaptic transmission specifically on L5/6 pyramidal neurons in the PL and IL cortices (Fig. 6) without affecting the L2/3 pyramidal neurons (Fig. S3). Compared to control animals, L5/6 pyramidal cells from stressed mice exhibited a remarkable increase in mEPSC frequencies (PL: Ctrl 1.182 ± 0.130, CS 2.020 ± 0.306, *p* = 0.0250; IL: Ctrl 0.503 ± 0.066, CS 1.163 ± 0.106, *p* < 0.0001) (Fig. 6b, g) and amplitudes (PL: Ctrl 11.124 ± 0.396, CS 12.848 ± 0.686; *p* = 0.0470; IL: Ctrl 10.043 ± 0.344, CS 11.921 ± 0.465, *p* = 0.0033) (Fig. 6c, h) in both PL and IL subregions. These alterations were accompanied by slower mEPSC decay kinetics (PL: Ctrl 4.632 ± 0.427, CS 5.718 ± 0.173, *p* = 0.0348; IL: Ctrl 4.542 ± 0.297, CS 5.686 ± 0.157; *p* = 0.0024) (Fig. 6d, i) with no change in rise times (PL: Ctrl 1.408 ± 0.045, CS 1.521 ± 0.080, *p* = 0.2465; IL: Ctrl 1.424 ± 0.047, CS 1.584 ± 0.064, *p* = 0.0583) (Fig. 6e, j). Additionally, L2/3 pyramidal cells from stressed mice presented a left-shifted curve of mEPSC amplitude (PL *p* < 0.0001; IL *p* = 0.0035) (Figs. S3c and h; right panel) indicative of predominantly lower amplitude excitatory events in both subregions, and a right-shifted curve of mEPSC interevent intervals (*p* < 0.0001) (lower frequency) only in the PL cortex (Fig. S3b; right panel). Moreover, a clear tendency towards decreased mEPSC amplitude (Ctrl 12.337 ± 0.848, CS 10.457 ± 0.343; *p* = 0.0690) (Fig. S3h; left panel) and frequency averages (Ctrl 3.334 ± 0.334; CS 2.523 ± 0.274; *p* = 0.0764) (Fig. S3b; left panel), was observed in L2/3 of the IL and PL, respectively. Reduced mEPSC decay kinetics (Ctrl 7.497 ± 0.200, CS 6.578 ± 0.341; *p* = 0.0346) and rise times (Ctrl 1.999 ± 0.105, CS 1.564 ± 0.084; *p* = 0.0051) were observed only in L2/3 pyramidal neurons from the IL subregion (Figs. S3i–j, d-e). Hence, our data demonstrate that CS has a tremendous impact on IL and PL cortices, increasing excitatory synaptic transmission onto L5/6 pyramidal cells in both cortical subregions. These observations may provide a mechanistic explanation for the increased glutamatergic excitatory inputs observed in striatal D1-MSNs after CS exposure.

**Fig. 7:**
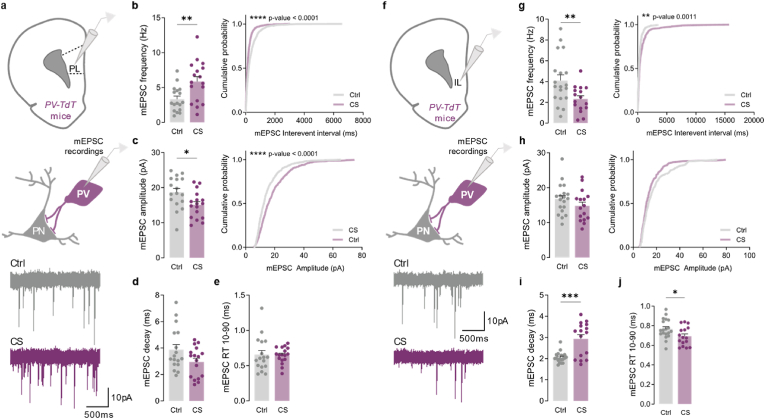
**Chronic stress differentially impacts excitatory synaptic transmission onto PV interneurons in L5/6 of prelimbic and infralimbic cortices**. (a) Example traces of miniature excitatory postsynaptic currents (mEPSC) recorded from tdTomato labeled parvalbumin (PV) interneurons from layer 5/6 (L5/6) in the prelimbic (PL) subregion, in control (Ctrl; grey) and stressed (CS; purple) mice. (b) Summary bar graphs (Ctrl *n* = 17 and CS *n* = 17 cells; **p = 0.0053) and cumulative probability curves (40 events per cell; ****p < 0.0001) show enhanced mEPSC frequency in L5/6 PV interneurons in the PL from stressed mice. (c) Summary bar graphs (Ctrl *n* = 17 and CS *n* = 17 cells; *p = 0.00171) and cumulative probability curves (40 events per cell; ****p < 0.0001) show decreased mEPSC amplitude in L5/6 PV interneurons in the PL from stressed mice. (d,e) Summary bar graphs (Ctrl *n* = 17 and CS *n* = 17 cells) show similar mEPSC decay kinetics and rise time (RT) in L5/6 PV interneurons in the PL from stressed mice. (f) Example traces of miniature excitatory postsynaptic currents (mEPSC) recorded from tdTomato labeled PV interneurons from L5/6 in the infralimbic (IL) subregion, in control (Ctrl; grey) and stressed (CS; purple) mice. (g) Summary bar graphs (Ctrl *n* = 19 and CS *n* = 16 cells; **p = 0.0077) and cumulative probability curves (15 events per cell; **p = 0.0011) show decreased mEPSC frequency in L5/6 PV interneurons in the IL from stressed mice. (h) Summary bar graphs (Ctrl *n* = 19 and CS *n* = 16 cells) and cumulative probability curves (15 events per cell) show no significant differences in mEPSC amplitude in L5/6 PV interneurons in the IL from stressed mice. (i,j) Summary bar graphs (Ctrl *n* = 19 and CS *n* = 16 cells) show slower mEPSC decay kinetics (***p = 0.0006) and reduced rise time (RT; *p = 0.0287) in L5/6 PV interneurons from the IL in stressed mice. All bar graphs are mean ± SEM; Two-sided Welch's unpaired *t*-test (b-e, g-j), and Kolmogorov-Smirnov test (b-c curves, g-h curves). Statistical details are shown in Sup.Table 1. (For interpretation of the references to colour in this figure legend, the reader is referred to the Web version of this article.)

Given that the activity of cortical pyramidal neurons is shaped by cortical PV interneurons (Markram et al., 2004; Sparta et al., 2014), we further asked whether glutamatergic synaptic transmission over cortical PV interneurons could also be impaired after CS. Accordingly, mEPSC recordings were obtained from PV interneurons in L5/6 of the PL and IL subregions (Fig. 7a, f). Surprisingly, our recordings revealed an opposite effect of CS between these two subregions. While PV interneurons from the PL region displayed enhanced mEPSC frequencies (Ctrl 3.330 ± 0.424, CS 5.856 ± 0.687; *p* = 0.0053) (Fig. 7b) with reduced amplitude (Ctrl 18.717 ± 1.069, CS 15.093 ± 0.897; *p* = 0.0171) (Fig. 7c), PV interneurons from the IL cortex presented a remarkable reduction in mEPSC frequency (Ctrl 4.116 ± 0.525, CS 2.325 ± 0.304; *p* = 0.0077) (Fig. 7g), slower mEPSC decay kinetics (Ctrl 2.060 ± 0.056; CS 2.935 ± 0.195, *p* = 0.0006) (Fig. 7i), and reduced rise time (Ctrl 0.768 ± 0.022, CS 0.692 ± 0.023; *p* = 0.0287) (Fig. 7j), in stressed mice.

Altogether, our data collectively suggests that CS selectively promotes activation of the striatal direct pathway by several possible parallel mechanisms: enhanced mPFC excitatory transmission, cortical “disconnection” from striatal PV interneurons, and increased excitation together with reduced inhibition over D1-MSNs, ultimately potentiating the recruitment of striatal direct pathway. Given that the direct pathway promotes the execution of motor actions, such pathological strengthening of the direct pathway may justify the emergence of motor symptoms observed in stress-related disorders.

## 4 Discussion

Despite the clear involvement of dorsomedial striatum circuits in stress-related disorders (Beyer et al., 2004; Hansen et al., 2002; Krishnan et al., 1992), the cell-specific alterations that occur after CS exposure are still poorly understood. Our study reveals important evidence suggesting that CS leads to a hyperactivation of the direct pathway by increasing excitatory synaptic transmission onto D1-MSNs and releasing them from the inhibitory influence of PV interneurons. Reduced striatal inhibition has been recently observed in CS and is hypothesized to emerge from dampened cortical excitation over striatal PV interneurons (Friedman et al., 2017). Our present data lend further support to this hypothesis by experimentally demonstrating that PV interneurons from stressed mice indeed receive weaker synaptic excitation after CS. Moreover, CS deeply remodels brain circuits in the IL and PL cortices, two prefrontal regions that strongly project to the dorsomedial striatum (Groenewegen et al., 1990; McGeorge et al., 1993; McGeorge and Faull, 1989). Our results specifically show that CS selectively impairs glutamatergic synaptic transmission onto pyramidal neurons and PV interneurons from layer 5/6, of both PL and IL, without affecting cortical neurons from layer 2/3. The layer selective effects of CS identified here are particularly interesting given what has been described in the literature: glutamatergic projections from cortical to striatal neurons arise mainly from pyramidal cells located on layer 5 (Jones et al., 1977; Kemp and Powell, 1970; Kitai et al., 1976; McGeorge and Faull, 1989; Oka, 1980; Royce, 1982; Tanaka, 1987; Veening et al., 1980), buttressing the idea of impaired prefrontal corticostriatal connectivity as a brain signature of stress exposure. Moreover, our morphological data revealed that D1-MSNs from stressed mice seem to have a more complex dendritic arborization (more intersections) from 140 to 260 μM from the soma (distal region of the dendrites), the primary site that receives glutamatergic projections from cortical structures (David Smith and Paul Bolam, 1990). However, it should be noted that PL and IL cortices project to several other brain regions apart from the striatum (Anastasiades and Carter, 2021). Furthermore, striatal neurons receive glutamatergic inputs not exclusively from the prefrontal cortex but also from the thalamus (Kreitzer and Malenka, 2008). Therefore, we cannot fully conclude that the reported alterations in glutamatergic synaptic transmission onto cortical neurons are the root of the striatal impairments. Rather, the increased glutamatergic transmission observed in D1-MSNs from CS mice can also arise from impaired thalamostriatal connectivity.

Although both PL and IL cortical circuits display clear functional defects after CS, the impact on the IL seems more pronounced when compared to the PL. Specifically, an increase in glutamatergic synaptic transmission onto pyramidal neurons, accompanied by a decrease of excitatory transmission onto PV interneurons, is robustly observed in the IL subregion, versus a moderate increase of excitatory inputs observed in the PL subregion for both neuronal types. Altogether, these results point to an overall robust overactivation of the IL. Noteworthy, the mPFC subregions studied here play opposite roles in controlling goal-directed and habitual actions: while PL controls goal-directed behaviour, IL supports the formation of habits (Amaya and Smith, 2018; Smith and Laiks, 2018). Concordantly, chronically stressed rodents tend to rely on habitual behavioural strategies (Dias-Ferreira et al., 2009; Friedman et al., 2017), seemingly corroborating our findings of robust overactivation of IL circuits under stress (Anastasiades and Carter, 2021; Kreitzer and Malenka, 2008). The selectivity of the D1 direct pathway circuit alterations we report here are also particularly relevant given what is known about striatum microcircuitry: PV interneurons innervate more D1 than D2 neurons (Gittis et al., 2010). This makes feedforward inhibition a more prominent feature of the D1 direct pathway (Gittis et al., 2010). Besides receiving more inhibitory projections from striatal PV interneurons, D1-MSNs are also more likely to receive glutamatergic inputs from the cortex, due to their extensive dendritic arbor (Gertler et al., 2008). Compared to D2-MSNs, D1 neurons have on average two more primary dendrites and are therefore estimated to be capable of receiving roughly 50% more glutamatergic inputs (Gertler et al., 2008). Thus, D1-MSNs are likely more vulnerable to pathological effects that arise from dysregulation of PV and cortical neurons. Accordingly, we observed stress-induced alterations in cortical neurons and striatal PV interneurons, as well as alterations in D1-MSNs only.

Altogether, we show that CS remodels cortical activity which may be responsible for triggering imbalanced levels of excitatory and inhibitory synaptic transmission in striatal circuits, culminating in increased excitation and reduced inhibition over direct pathway neurons only. We also show that CS not only decreases excitatory drive over striatal PV interneurons but also reduces PV excitability by hyperpolarizing their resting membrane potential, thus contributing to pathological disinhibition/hyperactivation of the striatal direct pathway. Notably, hyperactivation of the direct pathways has been previously hypothesized in OCD and Tourette syndrome (Ahmari et al., 2013; Burguière et al., 2015; Kalanithi et al., 2005; Monteiro and Feng, 2016b; Wang et al., 2009; Xu et al., 2016). Our observation of stress-induced direct pathway hyperactivation can thus provide a possible mechanistic explanation for stress-triggered OCD and potentially other relevant stress-induced disorders.

In summary, our data is well aligned with the general framework of striatum D1 motor function and with our previous work showing that stressed mice display increased motor locomotion (Rodrigues et al., 2022). We suggest a model where CS alters the excitatory synaptic drive of striatal neurons and releases the striatum from the inhibitory influence of PV interneurons, leading to hyperactivation of the D1 direct pathway of basal ganglia, causing long-lasting behavioural and physiological changes.

## CRediT authorship contribution statement

**Diana Rodrigues:** Conceptualization, the study, performed all experiments, and wrote the manuscript. **Patricia Monteiro:** Conceptualization, the study and critically revised the manuscript, All authors contributed to the article and approved the submitted.

## Declaration of competing interest

The authors declare that they have no conflict of interest.

## Data Availability

Data will be made available on request.
