# Supplementary material for: Chronic stress promotes basal ganglia disinhibition by increasing the excitatory drive of direct-pathway neurons
Source: Neurobiol Stress. 2023 Sep 22;27:100571. doi: 10.1016/j.ynstr.2023.100571 (PMC10540042; doi:10.1016/j.ynstr.2023.100571)
Supplement: Multimedia component 1 [file mmc1.pdf]

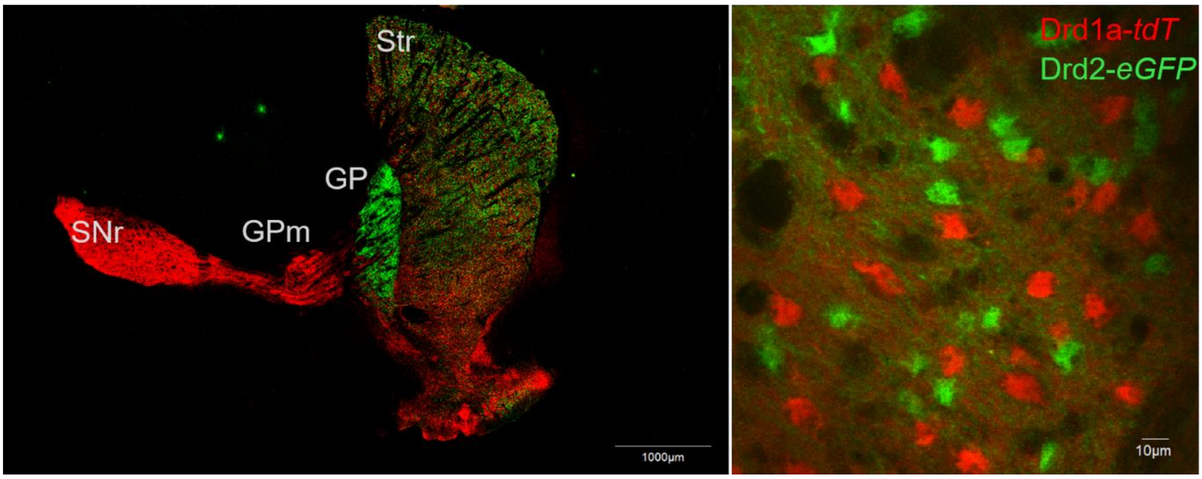

Figure S1. Drd1a-tdTomato mice and Drd2-EGFP mice faithfully label direct- and indirect-pathway projections from the striatum.

Drd1a-tdTomato mice crossed with Drd2-EGFP mice result in transgenic Drd1a-tdTomato:Drd2-EGFP mice. Although not useful for behaviour or electrophysiology experiments, these mice can be used for morphological visualization of both striatal direct and indirect pathways simultaneously. The image on the left shows a sagittal section where it is possible to observe direct pathway D1 neurons expressing tdTomato (red) and projecting directly to basal ganglia output nuclei (GPm, medial globus pallidus; SNr, substantia nigra pars reticulata); and indirect pathway D2 neurons expressing eGFP (green) and projecting to globus pallidus (GP). The image on the right shows a zoom-in detail of striatal D1 neurons soma expressing tdTomato (red) and D2 neurons soma expressing eGFP (green). Str: striatum

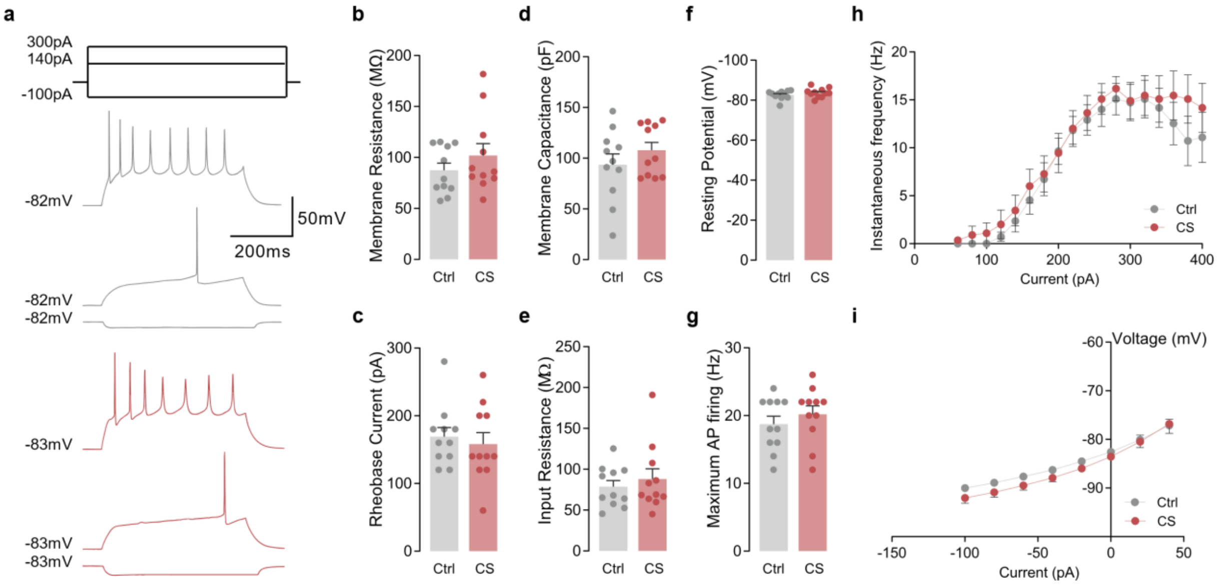

Figure S2. Chronic stress does not affect the intrinsic excitability of D1-MSNs.

(a) Representative current-clamp recordings from fluorescently labelled D1-MSNs in dorsomedial striatum region (DMS) of control (Ctrl; grey) and stressed (CS; red) mice.
(b) Membrane resistance (Ctrl *n* =11 and CS *n* =11 cells) measured with a 5mV pulse in voltage-clamp from -100 mV showed no significant alterations in D1-MSNs of stressed mice.
(c) Rheobase current (Ctrl *n* =11 and CS *n* =11cells) is not significantly altered in D1-MSNs of stressed mice.
(d) Membrane capacitance (Ctrl *n* =11 and CS *n* =11 cells) is not significantly altered in D1-MSNs of stressed mice.
(e) Input resistance (Ctrl *n* =11 and CS *n* =11 cells) measured with a -90 pA hyperpolarizing step from resting potential is not significantly different in D1-MSNs of stressed mice.
(f) Resting membrane potential (Ctrl *n* =11 and CS *n* =11 cells) is not significantly altered in D1-MSNs of stressed mice.
(g) Maximum action potential (AP) firing (Ctrl *n* =11 and CS *n* =15 cells) is not significantly altered in D1-MSNs of stressed mice.
(h) Action potential firing frequency (Hz) plotted as a function of injected current steps (Ctrl *n* =11 and CS *n* =11 cells). D1-MSNs’ intrinsic excitability showed no differences in stressed mice.
(i) Current-voltage plots (Ctrl *n* =11 and CS *n* =11 cells) recorded from D1-MSNs showed no differences in stressed mice.
All bar graphs are mean ± SEM; Two-sided Welch’s unpaired t-test (b-g), and two-way repeated-measures ANOVA (h-i). All statistical analyses are shown in Table 1.

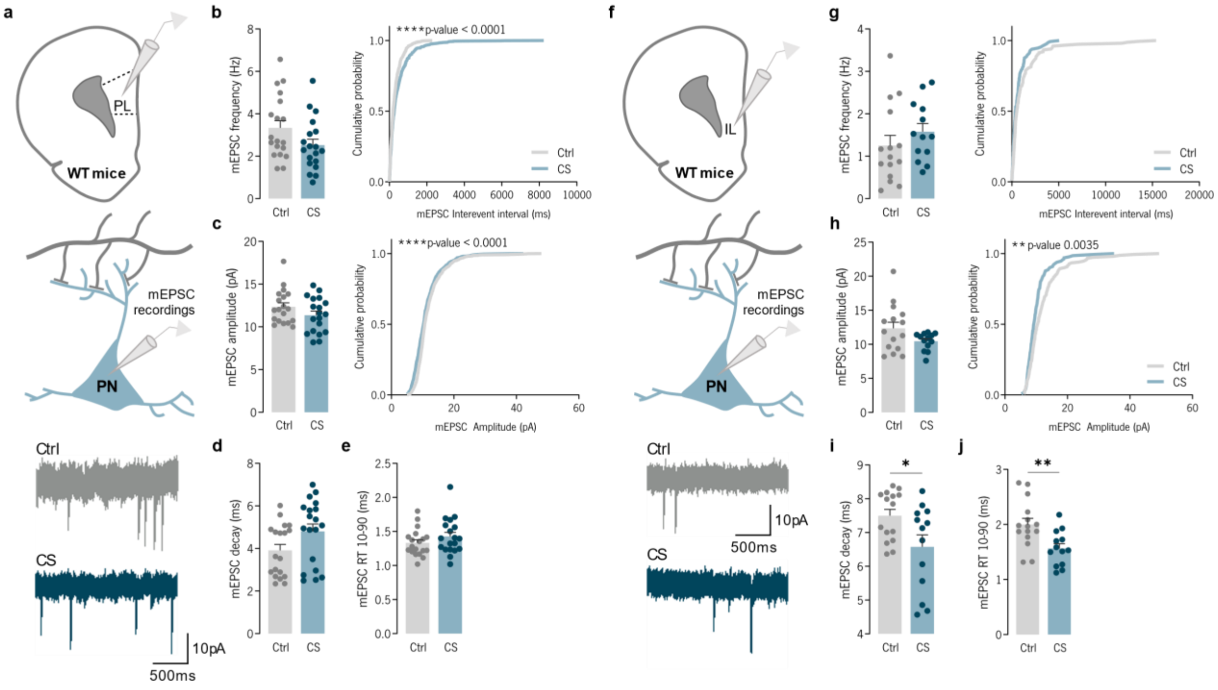

Figure S3. Chronic stress has little impact on excitatory synaptic transmission onto pyramidal neurons from layer 2/3 of prelimbic and infralimbic cortices.

(a) Example traces of miniature excitatory postsynaptic currents (mEPSC) recorded from layer 2/3 (L2/3) pyramidal neurons (PN) in the prelimbic (PL) subregion, in control (Ctrl; grey) and stressed (CS; blue) mice.
(b) Summary bar graphs (Ctrl *n* =19 and CS *n* =19 cells) show no significant difference in mEPSC average frequency (left panel). A right-shifted curve of mEPSC interevent interval can be observed in L2/3 pyramidal neurons in the PL of stressed mice (right panel; 50 events per cell; ****p<0.0001).
(c) Summary bar graphs (Ctrl *n* =19 and CS *n* =19 cells) show similar mEPSC average amplitude (left panel). A left-shifted curve of mEPSC amplitude can be observed in L2/3 pyramidal neurons in the PL of stressed mice (right panel; 50 events per cell; ****p<0.0001).
(d,e) Summary bar graphs (Ctrl *n* =19 and CS *n* =19 cells) show no alterations in mEPSC decay kinetics and 10%–90% rise time (RT) in L2/3 pyramidal neurons in the PL of stressed mice.
(f) Example traces of miniature excitatory postsynaptic currents (mEPSC) recorded from L2/3 pyramidal neurons (PN) in the infralimbic (IL) subregion, in control (Ctrl; grey) and stressed (CS; blue) mice.
(g) Summary bar graphs (Ctrl *n* =15 and CS *n* =13 cells) and cumulative probability curves (10 events per cell) show no alteration in mEPSC frequency in L2/3 pyramidal neurons in the IL of stressed mice.
(h) Summary bar graphs (Ctrl *n* =15 and CS *n* =13 cells) show identical mEPSC average amplitude (left panel). A left-shifted curve of mEPSC amplitude is observed in L2/3 pyramidal neurons in the IL of stressed mice (right panel; 10 events per cell; **p=0.0035)
(i,j) Summary bar graphs (Ctrl *n* =15 and CS *n* =13 cells) show reduced mEPSC decay kinetics (*p=00346) and 10%–90% rise time (RT; **p=0.0051) in L2/3 pyramidal neurons in the IL of stressed mice.
All bar graphs are mean ± SEM; Two-sided Welch's unpaired t-test (b-e, g-j), and Kolmogorov-Smirnov test (b-c curves, g-h curves). All statistical analyses are shown in Table 1.

Sup.Table 1 – Statistical Analysis

Figure	Measurement	*n*	Mean ± SEM	Statistical test and *p*-value
Fig.1a	D1-TdT positive cells morphology	Ctrl = 21/4 cells/mice<br>CS = 18/4 cells/mice	-	Two-way ANOVA with multiple comparisons *p=0.0115
Fig.1b	D1-TdT negative cells morphology	Ctrl = 14/4 cells/mice<br>CS = 14/4 cells/mice	-	Two-way ANOVA with multiple comparisons p=0.2052
Fig.2b left	mEPSC frequency (Hz)	Ctrl = 14/3 cells/mice<br>CS = 17/4 cells/mice	Ctrl 1.902 ± 0.290<br>CS 3.110 ± 0.284	Two-sided Welch's unpaired t-test, **p=0.0076
Fig.2b right	Cumulative Probability mEPSC interevent interval	30 events per cell	-	Kolmogorov-Smirnov test, ****p<0.0001
Fig.2c left	mEPSC amplitude (pA)	Ctrl = 14/3 cells/mice<br>CS = 17/4 cells/mice	Ctrl 12.740 ± 0.418<br>CS 13.739 ± 0.656	Two-sided Welch's unpaired t-test, p=0.2251
Fig.2c right	Cumulative Probability mEPSC amplitude	30 events per cell	-	Kolmogorov-Smirnov test, p=0.8266
Fig.2d	mEPSC decay time (ms)	Ctrl = 14/3 cells/mice<br>CS = 17/4 cells/mice	Ctrl 5.168 ± 0.496<br>CS 4.209 ± 0.255	Two-sided Welch's unpaired t-test, p=0.1131
Fig.2e	mEPSC rise time (ms)	Ctrl = 14/3 cells/mice<br>CS = 17/4 cells/mice	Ctrl 2.010 ± 0.067<br>CS 1.991 ± 0.084	Two-sided Welch's unpaired t-test, p=0.8663
Fig.2f	Excitatory Synaptic Drive	Ctrl = 14/3 cells/mice<br>CS = 17/4 cells/mice	Ctrl 24.658 ± 3.934<br>CS 44.676 ± 5.785	Two-sided Welch's unpaired t-test, **p=0.0100
Fig.2h left	mEPSC frequency (Hz)	Ctrl = 19/4 cells/mice<br>CS = 20/4 cells/mice	Ctrl 1.647 ± 0.120<br>CS 1.611 ± 0.147	Two-sided Welch's unpaired t-test, p=0.8526
Fig.2h right	Cumulative Probability mEPSC interevent interval	20 events per cell	-	Kolmogorov-Smirnov test, p=0.0733
Fig.2i left	mEPSC amplitude (pA)	Ctrl = 19/4 cells/mice<br>CS = 20/4 cells/mice	Ctrl 14.806 ± 0.336<br>CS 15.004 ± 0.480	Two-sided Welch's unpaired t-test, p=0.7430
Fig.2i right	Cumulative Probability mEPSC amplitude	20 events per cell	-	Kolmogorov-Smirnov test, p=0.8329
Fig.2j	mEPSC decay time (ms)	Ctrl = 19/4 cells/mice<br>CS = 20/4 cells/mice	Ctrl 3.397 ± 0.090<br>CS 3.250 ± 0.139	Two-sided Welch's unpaired t-test, p=0.3930
Fig.2k	mEPSC rise time (ms)	Ctrl = 19/4 cells/mice<br>CS = 20/4 cells/mice	Ctrl 1.916 ± 0.049<br>CS 1.859 ± 0.052	Two-sided Welch's unpaired t-test, p=0.4445
Fig.2l	Excitatory Synaptic Drive	Ctrl = 19/4 cells/mice<br>CS = 20/4 cells/mice	Ctrl 24.347 ± 1.789<br>CS 24.775 ± 2.729	Two-sided Welch's unpaired t-test, p=0.8992

Fig.3b left	mIPSC frequency (Hz)	Ctrl = 14/3 cells/mice<br>CS = 15/3 cells/mice	Ctrl 0.565 ± 0.075<br>CS 0.455 ± 0.037	Two-sided Welch's unpaired t-test, *p*=0.2180
Fig.3b right	Cumulative Probability mIPSC interevent interval	10 events per cell	-	Kolmogorov-Smirnov test, *p*=0.5403
Fig.3c left	mIPSC amplitude (pA)	Ctrl = 14/3 cells/mice<br>CS = 15/3 cells/mice	Ctrl 29.833 ± 2.601<br>CS 23.089 ± 1.315	Two-sided Welch's unpaired t-test, **p*=0.0369
Fig.3c right	Cumulative Probability mIPSC amplitude	10 events per cell	-	Kolmogorov-Smirnov test, **p*=0.0135
Fig.3d	mIPSC decay time (ms)	Ctrl = 14/3 cells/mice<br>CS = 15/3 cells/mice	Ctrl 9.036 ± 0.205<br>CS 8.063 ± 0.349	Two-sided Welch's unpaired t-test, **p*=0.0262
Fig.3e	mIPSC rise time (ms)	Ctrl = 14/3 cells/mice<br>CS = 15/3 cells/mice	Ctrl 2.545 ± 0.167<br>CS 2.519 ± 0.084	Two-sided Welch's unpaired t-test, *p*=0.8945
Fig.3f	Inhibitory Synaptic Drive	Ctrl = 14/3 cells/mice<br>CS = 15/3 cells/mice	Ctrl 17.047 ± 2.855<br>CS 10.444 ± 1.000	Two-sided Welch's unpaired t-test, *p*=0.0509
Fig.3h left	mIPSC frequency (Hz)	Ctrl = 15/3 cells/mice<br>CS = 15/3 cells/mice	Ctrl 0.459 ± 0.041<br>CS 0.589 ± 0.085	Two-sided Welch's unpaired t-test, *p*=0.1977
Fig.3h right	Cumulative Probability mIPSC interevent interval	10 events per cell	-	Kolmogorov-Smirnov test, *p*=0.2303
Fig.3i left	mIPSC amplitude (pA)	Ctrl = 15/3 cells/mice<br>CS = 15/3 cells/mice	Ctrl 32.727 ± 3.446<br>CS 32.415 ± 1.883	Two-sided Welch's unpaired t-test, *p*=0.9394
Fig.3i right	Cumulative Probability mIPSC amplitude	10 events per cell	-	Kolmogorov-Smirnov test, *p*=0.7232
Fig.3j	mIPSC decay time (ms)	Ctrl = 15/3 cells/mice<br>CS = 15/3 cells/mice	Ctrl 7.861 ± 0.398<br>CS 8.458 ± 0.195	Two-sided Welch's unpaired t-test, *p*=0.2085
Fig.3k	mIPSC rise time (ms)	Ctrl = 15/3 cells/mice<br>CS = 15/3 cells/mice	Ctrl 2.371 ± 0.151<br>CS 2.286 ± 0.113	Two-sided Welch's unpaired t-test, *p*=0.6669
Fig.3l	Inhibitory Synaptic Drive	Ctrl = 15/3 cells/mice<br>CS = 15/3 cells/mice	Ctrl 15.282 ± 2.140<br>CS 19.340 ± 3.061	Two-sided Welch's unpaired t-test, *p*=0.3039
Fig.4b left	mEPSC frequency (Hz)	Ctrl = 18/4 cells/mice<br>CS = 12/3 cells/mice	Ctrl 10.316 ± 0.580<br>CS 8.571 ± 0.791	Two-sided Welch's unpaired t-test, *p*=0.1012
Fig.4b right	Cumulative Probability mEPSC interevent interval	50 events per cell	-	Kolmogorov-Smirnov test, *p*=0.3038
Fig.4c left	mEPSC amplitude (pA)	Ctrl = 18/4 cells/mice<br>CS = 12/3 cells/mice	Ctrl 16.381 ± 0.479<br>CS 14.439 ± 0.446	Two-sided Welch's unpaired t-test, ***p*=0.0080
Fig.4c right	Cumulative Probability mEPSC amplitude	50 events per cell	-	Kolmogorov-Smirnov test, *p*<0.0001

Fig.4d	mEPSC decay time (ms)	Ctrl = 18/4 cells/mice<br>CS = 12/3 cells/mice	Ctrl 1.562 ± 0.040<br>CS 2.142 ± 0.214	Two-sided Welch's unpaired t-test, *p=0.0234
Fig.4e	mEPSC rise time (ms)	Ctrl = 18/4 cells/mice<br>CS = 12/3 cells/mice	Ctrl 0.601 ± 0.018<br>CS 0.651 ± 0.018	Two-sided Welch's unpaired t-test, p=0.0660
Fig.4f	Excitatory Synaptic Drive	Ctrl = 18/4 cells/mice<br>CS = 12/3 cells/mice	Ctrl 169.435 ± 11.191<br>CS 124.439 ± 12.124	Two-sided Welch's unpaired t-test, *p=0.0143
Fig.5b	Membrane Resistance (MΩ)	Ctrl = 19/4 cells/mice<br>CS = 15/3 cells/mice	Ctrl 93.738 ± 11.752<br>CS 123.268 ± 14.597	Two-sided Welch's unpaired t-test, p=0.1378
Fig.5c	Rheobase Current (pA)	Ctrl = 19/4 cells/mice<br>CS = 15/3 cells/mice	Ctrl 232.632 ± 17.374<br>CS 190.667 ± 17.473	Two-sided Welch's unpaired t-test, p=0.1086
Fig.5d	Membrane Capacitance (pF)	Ctrl = 19/4 cells/mice<br>CS = 15/3 cells/mice	Ctrl 47.309 ± 3.035<br>CS 50.069 ± 3.358	Two-sided Welch's unpaired t-test, p=0.5589
Fig.5e	Input Resistance (MΩ)	Ctrl = 19/4 cells/mice<br>CS = 15/3 cells/mice	Ctrl 101.237 ± 8.430<br>CS 116.193 ± 10.663	Two-sided Welch's unpaired t-test, p=0.2954
Fig.5f	Resting Potential (mV)	Ctrl = 19/4 cells/mice<br>CS = 15/3 cells/mice	Ctrl -74.891 ± 1.130<br>CS -78.091 ± 0.929	Two-sided Welch's unpaired t-test, *p=0.0416
Fig.5g	Maximum AP firing (Hz)	Ctrl = 19/4 cells/mice<br>CS = 15/3 cells/mice	Ctrl 84.421 ± 9.402<br>CS 88.400 ± 9.043	Two-sided Welch's unpaired t-test, p=0.7693
Fig.5h	IF curve	Ctrl = 19/4 cells/mice<br>CS = 15/3 cells/mice	-	two-way repeated-measures ANOVA, p= 0.8283
Fig.5i	IV curve	Ctrl = 19/4 cells/mice<br>CS = 15/3 cells/mice	-	two-way repeated-measures ANOVA, p=0.3886
Fig.6b left	mEPSC frequency (Hz)	Ctrl = 15/3 cells/mice<br>CS = 15/3 cells/mice	Ctrl 1.182 ± 0.130<br>CS 2.020 ± 0.306	Two-sided Welch's unpaired t-test, *p=0.0250
Fig.6b right	Cumulative Probability<br>mEPSC interevent interval	20 events per cell	-	Kolmogorov-Smirnov test, ****p<0.0001
Fig.6c left	mEPSC amplitude (pA)	Ctrl = 15/3 cells/mice<br>CS = 15/3 cells/mice	Ctrl 11.124 ± 0.396<br>CS 12.848 ± 0.686	Two-sided Welch's unpaired t-test, *p=0.0470
Fig.6c right	Cumulative Probability<br>mEPSC amplitude	20 events per cell	-	Kolmogorov-Smirnov test, ****p<0.0001
Fig.6d	mEPSC decay time (ms)	Ctrl = 15/3 cells/mice<br>CS = 15/3 cells/mice	Ctrl 4.632 ± 0.427<br>CS 5.718 ± 0.173	Two-sided Welch's unpaired t-test, *p=0.0348
Fig.6e	mEPSC rise time (ms)	Ctrl = 15/3 cells/mice<br>CS = 15/3 cells/mice	Ctrl 1.408 ± 0.045<br>CS 1.521 ± 0.080	Two-sided Welch's unpaired t-test, p=0.2465
Fig.6g left	mEPSC frequency (Hz)	Ctrl = 20/4 cells/mice<br>CS = 19/4 cells/mice	Ctrl 0.503 ± 0.066<br>CS 1.163 ± 0.106	Two-sided Welch's unpaired t-test, ****p<0.0001

Fig.6g right	Cumulative Probability mEPSC interevent interval	8 events per cell	-	Kolmogorov-Smirnov test, ****p<0.0001
Fig.6h left	mEPSC amplitude (pA)	Ctrl = 20/4 cells/mice<br>CS = 19/4 cells/mice	Ctrl 10.043 ± 0.344<br>CS 11.921 ± 0.465	Two-sided Welch's unpaired t-test, **p=0.0033
Fig.6h right	Cumulative Probability mEPSC amplitude	8 events per cell	-	Kolmogorov-Smirnov test, ****p<0.0001
Fig.6i	mEPSC decay time (ms)	Ctrl = 20/4 cells/mice<br>CS = 19/4 cells/mice	Ctrl 4.542 ± 0.297<br>CS 5.686 ± 0.157	Two-sided Welch's unpaired t-test, **p=0.0024
Fig.6j	mEPSC rise time (ms)	Ctrl = 20/4 cells/mice<br>CS = 19/4 cells/mice	Ctrl 1.424 ± 0.047<br>CS 1.584 ± 0.064	Two-sided Welch's unpaired t-test, p=0.0583
Fig.7b left	mEPSC frequency (Hz)	Ctrl = 17/4 cells/mice<br>CS = 17/4 cells/mice	Ctrl 3.330 ± 0.424<br>CS 5.856 ± 0.687	Two-sided Welch's unpaired t-test, **p=0.0053
Fig.7b right	Cumulative Probability mEPSC interevent interval	40 events per cell	-	Kolmogorov-Smirnov test, ****p<0.0001
Fig.7c left	mEPSC amplitude (pA)	Ctrl = 17/4 cells/mice<br>CS = 17/4 cells/mice	Ctrl 18.717 ± 1.069<br>CS 15.093 ± 0.897	Two-sided Welch's unpaired t-test, *p=0.0171
Fig.7c right	Cumulative Probability mEPSC amplitude	40 events per cell	-	Kolmogorov-Smirnov test, ****p<0.0001
Fig.7d	mEPSC decay time (ms)	Ctrl = 17/4 cells/mice<br>CS = 17/4 cells/mice	Ctrl 3.874 ± 0.380<br>CS 2.950 ± 0.269	Two-sided Welch's unpaired t-test, p=0.0642
Fig.7e	mEPSC rise time (ms)	Ctrl = 17/4 cells/mice<br>CS = 17/4 cells/mice	Ctrl 0.657 ± 0.058<br>CS 0.649 ± 0.027	Two-sided Welch's unpaired t-test, p=0.6565
Fig.7g left	mEPSC frequency (Hz)	Ctrl = 19/5 cells/mice<br>CS = 16/4 cells/mice	Ctrl 4.116 ± 0.525<br>CS 2.325 ± 0.304	Two-sided Welch's unpaired t-test, **p=0.0077
Fig.7g right	Cumulative Probability mEPSC interevent interval	15 events per cell	-	Kolmogorov-Smirnov test, **p=0.0011
Fig.7h left	mEPSC amplitude (pA)	Ctrl = 19/5 cells/mice<br>CS = 16/4 cells/mice	Ctrl 16.759 ± 0.985<br>CS 14.782 ± 1.066	Two-sided Welch's unpaired t-test, p=0.1955
Fig.7h right	Cumulative Probability mEPSC amplitude	15 events per cell	-	Kolmogorov-Smirnov test, p=0.0675
Fig.7i	mEPSC decay time (ms)	Ctrl = 19/5 cells/mice<br>CS = 16/4 cells/mice	Ctrl 2.060 ± 0.056<br>CS 2.935 ± 0.195	Two-sided Welch's unpaired t-test, ***p=0.0006
Fig.7j	mEPSC rise time (ms)	Ctrl = 19/5 cells/mice<br>CS = 16/4 cells/mice	Ctrl 0.768 ± 0.022<br>CS 0.692 ± 0.023	Two-sided Welch's unpaired t-test, *p=0.0287
